# The pattern of herbal medicines use for breastfeeding mother in Jogonalan, Klaten, Indonesia: a mini survey

**DOI:** 10.1186/s12906-023-04235-x

**Published:** 2023-11-07

**Authors:** Nutrisia Aquariushinta Sayuti, Nur Atikah

**Affiliations:** Department of Pharmacy, Health Polytechnic of the Ministry of Health of Surakarta, Jl. Ksatriyan 2, Danguran, South Klaten, 57425 Klaten, Central Java Indonesia

**Keywords:** Herbal medicine, Pattern of use, Breastfeeding mothers, Indonesia

## Abstract

**Background and objectives:**

Indonesian society extensively uses herbal medicine due to its abundant natural resources that have been utilized for generations for health purposes. Herbal medicine is also employed by specific community groups, such as breastfeeding mothers, to address issues like low milk production. However, there has yet to be much research conducted on its usage patterns in the Jogonalan District, Klaten Regency, Indonesia. It is crucial to understand this aspect to gain an overview of herbal medicine utilization in the community, thus preserving local’s healthy traditional culture and traditions and continuing their development. This study aims to describe the pattern of herbal medicine usage among breastfeeding mothers in the Jogonalan District, Klaten Regency, Central Java Province, Indonesia.

**Methods:**

The quantitative descriptive study conducted as a mini survey to breastfeeding mothers who used herbs, visited minimal three (3) times in integrated service posts for babies/toddlers in the Jogonalan sub-district and agree to overcome the research. Calculating the frequency distribution was used to analyze the study's findings. The advantages and disadvantages of these results are evaluated in relation to the findings of earlier investigations.

**Results and conclusions:**

The respondents were dominated housewives (84.4%), aged 20–35 years (71.1%), had secondary education (60.0%), and multiparous (66.7%). The daily use of *uyub-uyub* as a breast milk enhancer dominates the pattern of herbal medicine use. Papaya leaves, turmeric, and aromatic ginger are further herbal remedies. Nursing mothers’ health and fitness and breastfed children’s health is other advantages. The only adverse effects reported by three respondents (6.7%) were nausea and dizziness. Breastfeeding mothers can safely utilize evidence-based herbal medicines by increasing their knowledge, consulting with health professionals, and using them under supervision to preserve their use.

## Introduction

Traditional medicine or herbal medicine as complementary and alternative therapy has been widely used by the world community today. Strategies, support, and integration of herbal medicine have been developed by the World Health Organization (WHO) for adaptation into national health systems for WHO member [1]. Based on the 2017 Research on Medicinal Plants and Herbs, Indonesia has biological natural resources consisting of 2,848 species of medicinal plants with 32,014 medicinal herbs [2]. Husaini et al. discovered 74 plant species in Bantimurung-Bulusaraung National Park, effective as herbal medicine in treating 54 ailments [3]. The Basic Health Research results from 2010 to 2018 also showed that the user of traditional health providers had increased to 44.3% [2]. Rifka and Idris’s research stated that 73.8% of 163,259 respondents use herbal medicine [4]. Individuals use it for advantages like non-adverse effects, accessibility, and affordability [5]. In addition, other factors such as urban households, higher education, employment, economic status, and awareness of healthcare availability, also support increasing the use of herbal medicine [6].

Herbal medicine is a cultural heritage in Indonesia, widely used due to its diverse medicinal plants. Each region has a tradition of herbal medicine concoctions with local terminology, such as ‘*Jamu*’ on Java and ‘*Loloh*’ on Bali [7]. *Jamu* comes from the ancient Javanese language “*Jampi*” or “*usodo*” which means healing using potions, prayers or chants. The definition implies that *jamu* is not just a traditional health drink but is a variety of materials that can be used to treat health problems [8]. According to the Ministry of Health regulations, *Jamu* is defined as the terminology of Indonesian traditional medicine. *Jamu* is made from natural ingredients like plants, animals, minerals, and galenic preparations. It can be used for promotional, preventive, curative and palliative effort [9].

The diverse use of herbs for medicinal purposes dates back hundreds of years, as evidenced by historical relics, inscriptions, and temple reliefs. Ingredients for herbal medicine were found in letters, encyclopedia, personal notes, and oral narratives. *The Betaljemur Adammakna Primbon* is a Javanese encyclopedia detailing human life, including herbal disease recipes. It includes concotions like *cekok* for increasing appetite and *bobok*, *pilis*, *parem*, *boreh*, and *tapel* for external health complaints [10, 11]. Many medicin herbal concotions are also circulated and developed outside Java, for example ‘Sanrego’ for tonic drugs in Sulawesi, as well as ‘Lamatu’ which is even nicknamed Viagra from Donggala, Central Sulawesi because of its similarity in effect to Viagra (Sildenafil). The Dayak tribe in Kalimantan knows the ‘akar kuning’ used for generations to treat liver inflammation or hepatitis. ‘Akar kuning’ (Fibraurea chloroleuca) has a hepatoprotective effect [7].

Herbal medicine is also prevalent among breastfeeding mothers, as herbs are crucial for mothers and newborns [12]. The efficacy of herbal medicine to help alleviate heartburn, abdominal pain, birth canal pain, wrinkled skin, fear, and anxiety is essential for the mothers. while the efficacy of reducing breastfeeding interference and non-fluent milk is importants for the babies [13]. Various natural ingredients are used; namely, a mixture of *Sauropus* leaves with turmeric, *Zingiber zerumbet*, and tamarind, and also some herbs prepared from Sauropus leaves, banana blossoms, turmeric, Curcuma, ginger, aromatic ginger, tamarind, papaya leaves, betel leaves, cubeb, soybeans, moringa leaves, and almonds [14–18].

Indigenous knowledge on medicinal plants' effectiveness is supported by empirical evidence and scientific research on bioactive substances, such as scientification of *jamu* program, to demonstrate their safety and utility [19]. The review examines thirteen studies on six herbs as Galactagogue in Indonesia, including ginger and fenugreek. Four clinical trials were identified, with three related to ginger and one to fenugreek. Ginger contains constituents like gingerol, shogaol, paradol, and zingerone, which have a vasodilating effect. This effect is believed to promote milk production by enhancing blood flow to the breasts [20].

Central Java herbal medicines, including cabe puyang, uyup-uyup/gepyokan, beras kencur, kunyit asam, *and* paitan*,* are intangible cultural heritage in Indonesia, used for puerperium and breastfeeding [8]. Prastiwi stated that herbal medicine is safe for nursing mothers if used according to the rules of use [21]. Excessive use should be avoided to avoid side effects, including short-term, medium-term, and long-term problems. Short-term side effects include digestive disorders, medium-term side effects is liver function disorders, and the long-term side effect is impaired kidney function. Users must use hygienic, non-chemical, and preservative-free herbal medicine to ensure safety. Herbs made by the individual are safer, and the Indonesian Drug and Food Control Agency must have tested packaged herbs. Consultation with health personnel is necessary for the right type and dose of herbal medicine. Evaluation of the use of herbal medicine in nursing mothers by health practitioners also needs to be done to maximize the benefits and minimize the side effects of the herbs used [22].

**Fig. 1:**
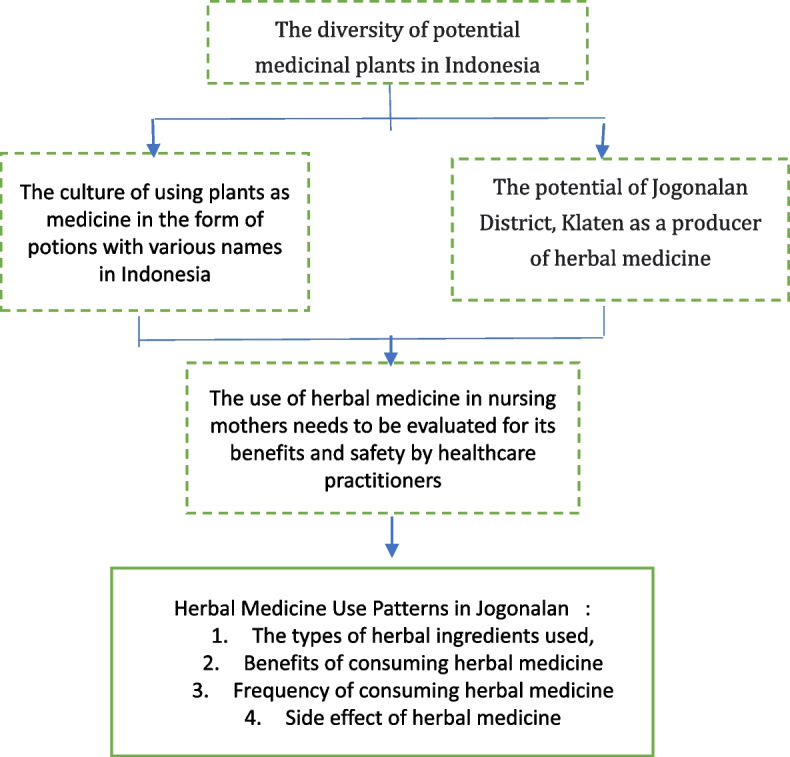
The theorical framework of mini survey.  : variable that is studied.  : not examined

The explanation above proves that research on herbal medicine usage patterns in each district or region still needs to be done in Indonesia. There has been no focused attention on breastfeeding mothers who use herbal medicines in Jogonalan, Klaten, even though the results of a survey conducted by the Central Statistics Agency stated that the percentage of Klaten residents who received traditional medicine in 2021 was 1.18% [23]. In addition, the results of the survey from Klaten’s statistics center showed that 32 herbal plant farmers in Jogonalan contribute 0.83 tons per hectare in the production of ‘chili pepper’ (Piper retrofractum) in Klaten [24]. There are four (4) herbal shops in Jogonalan. The number of herbal shops is the highest among the 26 sub-districts in Klaten district [25]. The data may indicate that the consumption of herbs in Jogonalan is relatively high. It is the reason why Jogonalan was chosen as the location of this survey. Research on patterns of herbal use in breastfeeding mothers in Jogonalan, Klaten is essential because it can provide an overview of the types of herbal ingredients used, frequency of consuming herbal medicines, benefits, and side effects experienced by users of herbal medicines. In addition, this research is helpful for health practitioners as an evaluation material to increase public interest in the use of effective and safe herbal medicine, especially for breastfeeding mothers, so the purpose of this study was to describe the pattern of use of herbal medicines in breastfeeding mothers in Jogonalan, Klaten, Indonesia. The survey focused on the types of herbal ingredients used, frequency of consuming herbal medicines, benefits, and side effects experienced by breastfeeding mothers. The survey results are discussed by comparing the results with theory, policy, and other related research results. The theoretical framework in this research can be seen in Fig. 1.

## Materials and methods

This research is a quantitative descriptive study as mini survey to describe patterns of herbal medicine use in breastfeeding mothers in Jogonalan, Klaten Regency, Indonesia. This study was approved by the Health Research Ethics Committee of Health Polytechnic of the Ministry of Health of Surakarta, Indonesia. No.LB.02.02/1.1/ 693.6 /2022. The variable in this study was a single variable, namely the pattern of use of herbal medicines by breastfeeding mothers in Jogonalan Klaten. The pattern of herbal use is operationally defined as the habit of consuming herbs in breastfeeding mothers consisting of the species of herbs used, the benefits of the herbs used, the frequency of herbal use, the occurrence of side effects and the types of side effects that breastfeeding mother has experienced. The selected respondents were breastfeeding mothers who visited minimum three (3) times of centers of integrated service for babies/toddlers in the Jogonalan sub-district and met the inclusion/exclusion criteria. The inclusion criteria were mothers breastfeeding children up to a maximum of one-year-old and using herbal medicines. While the exclusion criteria were breastfeeding mothers who were not willing to be respondents in this study.

The formula (1) was used to determine the number of respondents. Zα is 1.96 with a 95% confidence interval. d is the margin of error of 0.10. At the same time, p is the expected proportion based on the average prevalence of *jamu* (locally terminology of herbal medicine) use in breastfeeding mothers. We assumed the proportion of using herbal medicine was 13,96%. This assumption comes from data on 13,96% of the female population with exclusive breastfeeding and use herbs in the province of Central Java in 2014. The Indonesian Central Bureau of Statistics issued the data. It’s used because no proportion of breastfeeding mothers using herbal medicine in Jogonalan, Klaten and no research results were found on the proportion of exclusively breastfeeding mothers who use herbs regularly. q is the proportion of breastfeeding mothers who do not use herbal medicine (1-p). The minimum number of samples needed is 46 respondents, but only 45 respondents used herbal that met selected criteria (respondens with toddler maximum one years old and visited minimum three (3) times at centers of integrated service for babies/toddlers in the Jogonalan sub-district).

1 $$n=\frac{{Z}_{\alpha .P.Q}^{2}}{{d}^{2}}$$

This research used a questionnaire validated with Lawshe’s method of content validity. The Content Validity Ratio (CVR) is the foundation of the data analysis method used to evaluate the validity of the content. A data analysis method called CVR, created by Lawshe in 1975, is frequently used to assess content validity. By employing CVR to identify validation sheets based on question items, the Content Validity Index (CVI) may subsequently be computed [26].

The questionnaire was conducted and validated in January—March 2022. The research team developed the questionnaire. The unvalidated questionnaire consisted of 11 questions about respondent demographics (name, age, address, education, occupation, parturition statatus) and patterns of herbal medicine use (the type of herbs used, herbal benefits, frequency of herbal use, experience of side effects and types of herbal side effects experienced). Questions that are research variables are questions about the type of herbs used, herbal benefits, frequency of herbal use, experience of side effects and types of herbal side effects experienced. Open questions in the questionnaire were name, address, herbs used, benefits of herbs, and types of side effects. In contrast, the closed questions were education, occupation, parturition status, frequency of use, and side effect experience status. The questionnaire was validated by eight (8) panelists not part of the research team, namely two (2) public health experts, two (2) herbalists, two (2) midwives, and two (2) pharmacists.

The questionnaire used to collect content validity data was arranged with answer categories: ‘important’ with a score of 3, ‘valuable but not important’ with a score of 2, and not important with a score of 1. The answers given by the panelists were then analyzed by first converting the answers when answering ‘important’ and ‘valuable but not important’ will be worth 1, and if the answer is ‘Not important’ will be worth 0. After conversion, the panelist’s assessment for each item in the content validity questionnaire is calculated using CVR with the following formula: 2. CVR is the Content Validity Ratio. ne is the number of experts or panelists who answer important/valuable but not important, and n is the number of experts who validate.

2 $$\mathrm{CVR}=\frac{2ne}{n}-1$$

Question items in the questionnaire are declared accepted if the CVR value is equal to or more significant than the critical value. In contrast, the item will be rejected if the CVR value of the item is less than the critical value based on the number of panelists, as shown in Table 1. The Content Validity Index (CVI) value was calculated with formula 3 based on the CVR value obtained. CVI is the average of CVR values for validly answered question items. The CVI value will illustrate that each item in the questionnaire has good content validity [26].

**Table 1:** CVR critical value based on the number of panelists (one-tailed, α = 0.5) [26]

Number of panelists	CVR Critical Value
5	0,736
6	0,672
7	0,622
8	0,582

3 $$\mathrm{CVI}=\frac{\Sigma\;Valid\;CVR}{number\;of\;valid\;items}$$

Data collection was carried out from April 2022 to July 2022. Random selection was not done in the recruitment of respondents. Data for prospective respondents who met the criteria were obtained from data at centers of integrated services for babies/toddlers. The recruitment process of respondents was carried out as follows:. The integrated service post is under the coordination of midwives. One of the health programs implemented at the integrated service post examines the growth of babies and toddlers in the program, which also involves breastfeeding mothers as program participants. The midwives who work at the integrated service post gave recommendations to breastfeeding mothers who met the inclusion criteria as potential respondents in the survey. The research team screened the breastfeeding mothers as potential respondents by gathering them at the integrated service post by asking if they used herbal medicine during breastfeeding. The integrated service post was chosen because the location of the place is closer to the prospective respondents.

If prostpective respondents use herbal medicine during breastfeeding, then it was explained that they can be voluntary respondents in the survey. The research team explained the activity of surveying herbal medicine usage patterns in Jogonalan Klaten, volunteering to participate in research, research procedures, obligations as respondents, research benefits, and research confidentiality. The explanation about volunteering to be a respondent is an explanation of the freedom of prospective respondents to participate in the survey. If the prospective respondent decides to participate, the respondent is free to resign or change his mind at any time without incurring fines or sanctions. The research procedure is the explanation of filling out the questionnaire or interview according to the questionnaire if the respondent wants, filling in the identity, signing the consent form, and answering the questions according to the questionnaire. The obligation as a respondent explains that the respondent must answer the questionnaire, and if there is anything unclear, they can ask the research team. The benefits of the research explain that the research is expected to improve the public’s understanding of herbs used in breastfeeding mothers. The explanation about the confidentiality of the research is that all information related to the identity of the respondents will be kept confidential and only known by the researcher.

Prospective respondents were asked for their agreement and willingness to participate in the research. If they agreed to be research respondents, they filled out and signed a ‘written consent form after explanation’. Suppose they signed a consent form indicating their consent to participateas respondents in this survey. Data of survey was collected through face-to-face interviews between the research team and the respondents or by the respondents filling out the questionnaire. Respindents are guaranteed confidentiality and can opt out anytime when answering questions.

The data analysis was conducted in August 2022 until November 2022. The analysis was univariate, using frequency distribution for research findings. Frequency distribution was reported descriptively, narrated, and discussed. The advantages, as well as disadvantages of the results of this study, were evaluated concerning the findings of previous studies. Conclusions were drawn to answer research objectives and investigate suggestions or further research needed to increase the use of herbal medicines.

## Results

Table 2 showed the result of validation. The CVR result was 0.250 to 1.000. Questions is valid if the CVI is more than 0.582 [26]. Questions about the names and addresses of respondents were considered invalid because the data could already be obtained from the ‘inform consent form’ (CVR = 0.250). Nine (9) questions with a CVR value of more than a critical value (0.582) have a CVI value 0.972. As a result, the validated questionnaire has nine questions: age, education, occupation, parturition status, herbs used, herbal benefits, frequency of herbal use, side effect experience status, and side effects type. The questionnaire used in the research with nine questions about age, education, occupation, parturition status, herbs used, herbal benefit, frequency of herbal use, side effect experience status and side effect type.

**Table 2:** Validation results of questioner

NO	Question about	CVR	Validation criteria	CVI of valid CVR	Validation Criteria
1	Name	0.25	Invalid	0.972	Valid
2	Age	1	Valid		
3	Addresse	0.5	Invalid		
4	Education	1	Valid		
5	occupation	1	Valid		
6	parturition status	1	Valid		
7	herbs used	1	Valid		
8	herbal benefits	1	Valid		
9	frequency of herbal use	1	Valid		
10	side effect experience status	0.75	Valid		
11	side effects type	1	Valid		

The characteristics of the age, education, occupation, and parturition status of the respondents are shown in Table 3. The ages indicate that most respondents were 20–35 (71.1%). Most respondents with secondary education (senior high school) worked as housewives. Multiparous more than primiparous.

**Table 3:** Characteristics of respondents (*n* = 45)

Characteristics	n (%)
Age	
a. < 20 years	2 (4.4)
b. 20 – 35 years	32 (71.1)
c. > 35 years	11 (24.4)
Educational Stage	
a. Primary	15 (33.4)
b. Secondary	27 (60.0)
c. Academy or above	3 (6.7)
Type of Work	
a. Housewife	38 (84.4)
b. Civil Servants	1 (2.2)
c. Private employe	6 (13.3)
Parturition Status	
a. Primiparous	15 (33.3)
b. Multiparous	30 (66.7)

Table 4 shows the pattern of herbal medicine use among nursing mothers in Jogonalan Klaten, Indonesia. It showed that the majority of respondents use *Uyub-uyub* to maintain their health. *Uyub-uyub* is a herbal concoction indicated to increase the volume of breast milk. The herbs combine Sauropus leaves, papaya, and another herb to boost breast milk. Papaya leaves were the second herb that nursing mothers widely used.

**Table 4:** The pattern of herbal medicines use during breastfeeding in Jogonalan, Klaten, Indonesia (*n* = 45)

Variables	n (%)
Herbs	
a. *Uyub-uyub* (Javanesse breastmilk booster formula)	15 (33.3)
b. Papaya leaves	12 (26.7)
c. *Jamu* (Indonesian Traditional Medicine)	7 (15.6)
d. *Jamu Gendong* (Indonesian Traditional medicine marketed by artisan herbs)	6 (13.3)
e. Kunir asem (Combination of Tumeric and tamarind)	4 (8.9)
f. *Kencur* (*Kaempferia galanga* Linnaeus)	4 (8.9)
g. Kunyit (Tumerics/ *Curcuma Longa* L)	3 (6.7)
h. *Paitan* (Bitter herbs)	2 (4.4)
i. Betel leaves (*Piper betle* L.)	1 (2.2)
j. *Brotowali* (*Tinospora caulis* L)	1 (2.2)
Benefits of herbs	
a. Breast milk booster	34 (75.6)
b. Refreshing body	7 (15.6)
c. Healthy body	4 (8.9)
d. Increased breast milk	4 (8.9)
e. Breast milk becomes fresh	2 (4.4)
f. Increased appetite	1 (2.2)
g. Increased body endurance	1 (2.2)
h. Babies become healthier	1 (2.2)
i. Babies get fat	1 (2.2)
Frequency of consuming herbs	
a. Daily	41 (91.1)
b. Weekly	11 (24.4)
c. Monthly	3 (6.7)
Experiencing side effects	
a. Yes	3 (6.7)
b. No	42 (93.3)
Kind of side effects	
a. Nausea	1 (1.1)
b. Dizziness	1 (1.1)
c. No answer	1 (1.1)

The benefits of herbs for breast milk included expediting, increasing, and refreshing breast milk. In addition, respondents also felt that herbs made their bodies healthier and fitter and increased their appetite. Respondents also informed that when the baby will suckle more breast milk to become healthier and fatter if they consume herbal medicine. Table 5 shows the benefits of using each herbal medicine. Uyub-uyub, papaya leaves, jamu gendong, turmeric, paitan, betel leaves, and brotowali are widely used as milk boosters. Herbal users feel the benefit of increasing the amount of breast milk, which becomes fresh. Kencur is used to refresh the body.

**Table 5:** Benefits of each kinds of herbs by respondents (*n* = 45)

Benefit (n (%))	Breast milk booster	Refresh-ing body	Healthy body	Increas-ed breast milk	Breast milk becomes fresh	Increas-ed appetite	Increased body endurance	Babies become healthier	Babies get fat	N (%)
**Kinds of herbs**										
*Uyub-uyub* (Javanesse breastmilk booster formula)	13 (28.9)	NA	NA	1(2.2)	NA	NA	1 (2.2)	NA	NA	15 (33.3)
Papaya leaves	8 (17.8)	1 (2.2)	1 (2.2)	1 (2.2)	NA	NA	NA	NA	1 (2.2)	12 (26.7)
*Jamu* (Indonesian Traditional Medicine)	1 (2.2)	1 (2.2)	NA	2 (4.4)	2 (4.4)	NA	NA	1 (2.2)	NA	7 (15.6)
*Jamu Gendong* (Indonesian Traditional medicine marketed by artisan herbs)	6 (13.3)	NA	NA	NA	NA	NA	NA	NA	NA	6 (13.3)
Kunir asem (Combination of Tumeric and tamarind)	1 (2.2)	2 (4.4)	NA	NA	NA	1 (2.2)	NA	NA	NA	4 (8.9)
Kencur (Kaempferia galanga Linnaeus)	1 (2.2)	2 (4.4)	1 (2.2)	NA	NA	NA	NA	NA	NA	4 (8.9)
Kunyit (Tumerics/ *Curcuma Longa* L)	1 (2.2)	1 (2.2)	1 (2.2)	NA	NA	NA	NA	NA	NA	3 (8.9)
*Paitan* (Bitter herbs)	1 (2.2)	NA	1 (2.2)	NA	NA	NA	NA	NA	NA	2 (4.4)
Betel leaves (*Piper betle L.)*	1 (2.2)	NA	NA	NA	NA	NA	NA	NA	NA	1 (2.2)
*Brotowali* (*Tinospora caulis* L)	1 (2.2)	NA	NA	NA	NA	NA	NA	NA	NA	1 (2.2)
Total (n(%))	34 (75.6)	7 (15.6)	4 (8.9)	4 (8.9)	2 (4.4)	1 (2.2)	1 (2.2)	1 (2.2)	1 (2.2)	55 (122.2)^a^

^a^The columns do not add up to 100% because the respondent wrote the number of herbs more than 1

*NA* No available

The majority of respondents consume herbs with daily doses. Table 6 shows that the majority of *kunir asem* consumption frequency is weekly. Usually, they taken one (1) glass (200 ml) once a day. Only three (3) respondents experienced side effects when using herbs. One (1) respondent answered side effects in the form of nausea, one (1) respondent experienced dizziness, and one (1) respondent did not answer what side effects she felt. Three (3) respondents who experienced side effects from the consumption of *uyub-uyub*, jamu and *kunir asem*.

**Table 6:** Frequency of uses of each kind of herbs by respondents (*n* = 45)

Frequency (n (%))	Dayli	Weekly	Monthly	Total (n (%))
**Kinds of herbs**				
*Uyub-uyub* (Javanesse breastmilk booster formula)	12 (26.7)	3 (6.7)	NA	15 (33.3)
Papaya leaves	8 (17.8)	3 (6.7)	1 (2.2)	12 (26.67)
*Jamu* (Indonesian Traditional Medicine)	5 (11.1)	2 (4.4)	NA	7 (15.6)
*Jamu Gendong* (Indonesian Traditional medicine marketed by artisan herbs)	5 (11.1)	NA	1 (2.2)	6 (13.3)
Kunir asem (Combination of Tumeric and tamarind)	1 (2.2)	2 (4.4)	1 (2.2)	4 (8.9)
Kencur (*Kaempferia galanga Linnaeus*)	3 (6.7)	1 (2.2)	NA	4 (8.9)
Kunyit (Tumerics/ *Curcuma Longa* L)	3 (6.7)	NA	NA	3 (6.7)
*Paitan* (Bitter herbs)	2 (4.4)	NA	NA	2 (4.4)
Betel leaves (*Piper betle L.)*	1 (2.2)	NA	NA	1 (2.2)
*Brotowali* (*Tinospora caulis* L)	1 (2.2)	NA	NA	1 (2.2)
Total (n (%))	41 (91.1)	11 (24.4)	3 (6.7)	55 (122.2)^a^

^a^The columns do not add up to 100% because the respondent wrote the number of herbs more than 1

*NA* No available

## Discussions

The majority of respondents’ age were 20 – 35 years old. Aged 20–35 years are included in the healthy reproductive group because the reproductive organs are ready to carry out the reproductive process. It’s also related to psychological and mental maturity in supporting exclusive breastfeeding for infants [27]. The majority of education levels of respondents are secondary education. Education is one of the factors that influence the decision of breastfeeding mothers to use herbs. Breastfeeding mothers with high education tend to have better health literacy, so they often use non-conventional modes of care. Good health literacy makes breastfeeding mothers active in seeking additional healthcare methods beneficial to their health conditions [28]. In Indonesia, non-conventional modes of care is herbal medicine, complementary therapies and non-medical physical therapy. Fifty six (56) hospitals in 18 provinces in Indonesia have provided non-conventional therapies such as alternative medicine or traditional herbal medicine. The government issued a set of legal policies to support unconventional therapy, including Regulation of the Minister of Health of the Republic of Indonesia number. 003 in 2010 concerning the Indonesian herbal medicine, scientification of jamu program (saintifikasi jamu). The regulates is for scientific evidence of traditional medicines through service-based research (dual system), as well as the use of traditional medicines for promotive and preventive (health and fitness maintenance), curative (treating disease), and palliative (improving quality of life) purposes in Indonesia [29].

The majority of respondent were housewives. Millinga et al. [28] stated that the type of work did not affect the decision to use herbs in breastfeeding mothers. The kind of work, whether formal or informal, did not affect the use of herbs in nursing mothers [30]. The research also declared similar results by Vardanjani et al., which stated that there was no difference in the type of work between breastfeeding mothers who use herbs and those who do not [31]. Employment status is linked to an individual’s economic condition. The housewives relying on their husbands for income. Their financial dependence leads them to follow their husbands’ choices, including herbal medicine use [32]. The low income level decreases the ability to seek treatment, so herbal medicine ines are preferred because they are cheaper [33].

The majority of respondents’ educational stage were secondary education. Adyasa and Meiyanti stated that the level of education is related to the ability to receive or remember information or knowledge. Someone with higher education tends to believe more in rational and objective thinking so they prefer modern medicine with complete clinical trials to herbs. Higher education creates a critical attitude in receiving information so that it reconfirms the information. Insights about the effectiveness and safety of herbal medicines are more numerous and accurate so that their use can be more responsible [33].

The results showed that multiparaos dominated more than primiparous. Breastfeeding mothers with primiparous status are usually less experienced in dealing with health problems postpartum [31]. The condition makes them visit health facilities. The anxiety level of mothers with primiparous is also higher than that of multiparous because primiparous mothers still need to adapt to their situation after the birth process [34]. Therefore, mothers with primiparous status can overcome their anxiety related to breast milk production by consuming herbs. The use of these herbal medicines has prevented the majority of mothers with primipara status from having breastfeeding problems [31].

The pattern of use of herbal medicines in breastfeeding mothers in Jogonalan, Klaten, showed that *uyup-uyup* or *uyub-uyub* was the most widely used herb. It is also proven from the results of the research that many respondents use uyub uyub as a breast milk booster. *Uyub-uyub* is a herbal formula for fluent breastmilk in Central Java. The herbal composition found in *uyub-uyub* includes aromatic ginger, turmeric, *temu giring* (*Curcuma heyneana* Valeton & v. Zijp), *temulawak* (Javanese ginger (*Curcuma zanthorrhiza* Roxb.)) and *katuk* leaves (Sweet leaf/Star Gooseberry (*Sauropus androgynus*)). In addition to facilitating breastfeeding, the contents of aromatic ginger and *temu giring* can also warm the body and provide a sense of calm so that the mother's psychological condition becomes stable. As a result, the production of the hormone oxytocin will be stimulated, which in turn can stimulate prolactin to continue producing breast milk. *Uyup-uyup* is usually used every day if there is a problem with the flow of breast milk. Use starts from the first week after delivery to the fourth week. Fresh herbs are boiled and then the boiled water is drunk after cooling. The volume of use of uyup-uyup is one glass per day. Some information from parents stated that *uyup-uyup* is used twice a day one glass (morning and night) in the first week after giving birth. This dose is reduced to one glass a day after breastfeeding smoothly or in the sixth week after giving birth [21].

In 2014, Kumalasari et al. [35] studied *uyup-uyup* using the static group comparison method on 30 breastfeeding mothers in the Kemangkon Health Center, Purbalingga district. It using an accidental sampling technique and a questionnaire instrument. The results showed that of the 15 respondents who consumed *uyup-uyup*, 100% had smooth breastfeeding, while the 15 respondents who did not consume it, only 2 (13.3%) had smooth breastfeeding. The p-value of the Mann Whitney U test was 0.000 < α 0.05, which means that there was an effect of giving *uyup-uyup* to the smooth release of milk in postpartum mothers.

Prastiwi [21] reported the side effects of *uyup-uyup* in the form of dizziness. It is due to the increase in blood pressure in nursing mothers. The flavonoids in *uyup-uyup* have a hypotensive effect with the mechanism of action inhibiting the activity of ACE and diuretic. The finding of increased blood pressure contradicts the mechanism of action of flavonoids. Health workers recommend to reduce the consumption of herbs to once a day. The suggestion was successful in normalizing the blood pressure of *uyup-uyup* consumers.

Papaya leaf was the second herb that respondents mainly used as a milk booster, to refresh the body, make body healthy and make the baby fat. Papaya (*Carica papaya* L) is a simplicia that can increase the bulk of breast milk secretion. Pebrianthy et al. [36] stated that giving papaya leaf decoction is an effort to improve nutrition for breastfeeding mothers. Research conducted on 27 breastfeeding mothers in Padangsidempuan showed that papaya stew affected increasing milk production in nursing mothers (*p*-value = 0.001, (*p* < 0.05)) [36].

Papaya leaves contain quercetin which can activate the prolactin hormone that helps increase breast milk. Daily administration of papaya leaf juice to nursing mothers for seven days can increase the mother's prolactin hormone and the baby's weight. The average prolactin hormone increased in breastfeeding mothers is 19.59 ng/ml, while the baby's weight gain was 165 g. The results of the Wilcoxon test for increased levels of the hormone prolactin *p*-value 0.047 < 0.05 and for increased newborn weight *p*-value 0.009 < 0.05 [37].

Concoctions from papaya leaves are usually made fresh by grinding fresh papaya leaves and warm water and filtering to extract the juice. When the herb will be consumed, the preparation is recenter paratus or made new. The taste is very bitter, making this herb only consumed in the morning 200 ml. Its consumption during breastfeeding is usually only for five days and sometimes stops if the breast milk is smooth even if the frequency of use is made once a week or once a month because of its bitter taste. Papain in papaya leaves causes allergies so people with papain allergy should be careful when consuming papaya leaves [38].

Turmeric was used as a single herbal or in combination with tamarind. The benefits of turmeric for nursing mothers are reducing pain, lowering blood pressure, reducing inflammation, and increasing blood circulation. It is also proven in the answers of the majority of respondents that the benefit of turmeric for nursing mothers is refreshing the body. In addition, by stimulating the anterior pituitary to produce more prolactin, which increases milk production, some chemical components in these herbs have an estrogenic impact that may improve milk production [39]. Turmeric that has been washed clean with running water, ground with water and then boiled and filtered. The infusa is consumed 200 ml every day for 7 days postpartum [38].

There is no information on the excretion of any turmeric constituents into breastmilk. In a tiny trial, babies who consumed milk with turmeric had no negative consequences. Even at large doses, turmeric is typically well tolerated, however gastrointestinal side effects such nausea and diarrhea as well as allergic reactions have been documented. Patients using antiplatelet medications such as warfarin and turmeric may experience increased bleeding risk. Curcumin inhibits milk production in lactating mammary epithelial cells when tested in vitro. Due to a paucity of information, turmeric at doses greater than those used as a flavour in food should be avoided when nursing [40].

Tamarind and turmeric is an alternate method of treating wounds. It has been established that turmeric and tamarind have anti-inflammatory, antioxidant, anticarcinogenic, anti-infective, analgesic, and anticoagulant properties. In addition, Turmeric and Tamarind work in different phases to accelerate the migration of epithelial cells under the wound, thereby accelerating wound closure. A quasi-experimental study with a group post-test-only design was conducted to test the efficacy of tamarind turmeric as a healing agent for peritoneal wounds at the Ngesrep and Srondol Health Centers in Semarang City, Central Java, Indonesia. The results stated that fourteen (14) breastfeeding mothers who consumed tamarind turmeric showed an average wound healing time in the intervention group was 6.25 days. The average wound healing was 8.57 days for 14 breastfeeding mothers in the control group. *P*-value 0.000 (< 0.05) indicated a significant effect of consumption of tamarind turmeric on the healing time of perineal wounds [41].

Turmeric tarmarind is made by boiling turmeric, tamarind, salt, brown sugar and sugar with water until the sugar dissolves. Cool and strain, then pound and filter the resulting juice. Then store in a clean bottle for daily consumption (200 ml every morning) [42]. The tamarind fruit is considered harmless by the WHO, but it should not be consumed by those with specific medical disorders or allergies. It contains high sugar content, making it a risk for diabetics. Tamarind is also a potent purgative, so those with kidney or gallbladder issues should avoid it. Consult a nutritionist or healthcare professional to establish a personal upper limit for tamarind consumption [43]. Tamarind also contains a lot of acid. Overconsumption can cause acidity by raising the acid level in the gastrointestinal tract. No sufficient to determine whether tamarind consumption is safe for pregnant and nursing mothers. If taking anti-hypertensive or anti-diabetic medicine, it is best to eat tiny amounts of tamarind fruit extract. Some might advise to avoid it. However, none of these assertions have been validated [44].

*Kencur* (*Kaempferia galanga L*) is ingredients of *uyub-uyub* [45]. Its traditional use is as an expectorant and mucus thinner to relieve nasal congestion, stomach upset, diabetes, rheumatoid arthritis, fever, and carminative. The research data states that many respondents use kencur as a refreshing body. *Kencur* rhizome contains primary metabolites such as protein, fiber, and minerals (potassium, phosphorus, magnesium, zinc, cobalt, iron, manganese, and nickel). The secondary metabolite in *kencur* is kaempferol which functions as an antioxidant. The combination of kaempferol and cinnamic in *kencur* can increase the vasodilatory effect of *kencur*. In addition, the essential oil in ethyl p-methoxycinnamate (EPMC) can produce anti-inflammatory effects [46]. Kencur rhizome is to boil 5 g of kencur with 200 ml of water, consumed thrice daily. Although information regarding safety and efficacy in pregnancy and lactation is lacking, this herb is contraindicated in allergic conditions, pregnancy and chronic stomach disorders because the side effect is a burning sensation in the stomach if not used according to the recommended dosage [47].

*Paitan*, derived from the word ‘*pahit*’, which means bitter, is a herbal medicine made from bitter herbs (*Andrographis paniculata* Ness) and *brotowali* (*Tinospora crispa*), pule (*Alstonia scolaris* L. R. Br.), *widoro laut* (*Strychnos ligustrina*), some add fennel (*Foeniculum vulgare*) as an additional recipe. Data shows that respondents use paitan as a breast milk booster. The herb symbolizes an adult life that is bitter but must be lived. From this name, it can be concluded that the taste of this herb is bitter and is efficacious for relieving itching, such as cleaning the blood or preventing allergies [48]. The mechanism of action as a breast milk booster has never been studied. Its effectiveness as a breast milk booster may be related to its benefits in overcoming lack of appetite, pain, and dizziness so that it can reduce the mother's stress and indirectly increase the amount of breast milk [49]. Paitan is rarely used in nursing mothers due to contraindications in pregnancy, breastfeeding, allergies, and children. It can cause stomach discomfort, nausea, vomiting, loss of appetite, and allergies. It can interact with immune suppressing drugs, blood thinners, and isoniazid, making it advisable to avoid its use [47].

Data shows that betel leaf is used as a breast milk booster. Betel leaves have chemical contents of essential oil such as hydroxy chavicol, cavibetol, estragole, eugenol, methyl eugenol, and carvacrol. The essential oil functions as an antibacterial, antifungal, antioxidant, and antiseptic [50]. Betel leaves are boiled in clean water, cooled, and then used to wash the female genital organs. Betel leaf decoction can also be taken to treat Leucorrhoea/Fluor albus. Betel leaves (*P. betel*), turmeric leaves (*Curcuma longa*), and ginger rhizome (*Zingiber officinale*) were boiled. It consumed for 40 days after the mother delivered labor. The benefit is to accelerate postpartum wound healing. Betel leaf pounded with papaya root and areca nut (*Areca catechu*). Then it is rubbed on the breast accompanied by massage from back to front, which helps stimulate the release of breast milk [51].

Betel leaf water extract is safe for up to 1,000 mg/kg body weight, with no negative effects on blood image, biochemistry, or enzymes. However, chewing betel with gambir and areca nuts, which pregnant women consume, raises concerns. A study in Papua New Guinea found that chewing betel while pregnant increased the likelihood of negative outcomes for the unborn child, similar to when pregnant women smoke or drink alcohol. Lower birth weight led to more early deliveries and shorter births. Further scientific research is needed to establish the efficacy of betel’s use for post-partum care [51].

The data indicates the term *jamu* and *jamu gendong* without specifying the herbal used. Both are used as breast milk boosters, increase milk production, make milk fresh, refresh the body, and make the baby healthy. Jamu is the local term for traditional medicine in Central Java, Indonesia. They do not know its content because they buy it by mentioning their needs to the seller. Indonesia categorizes traditional medicines into three groups based on clinical trials availability: phytopharmaca, standardized herbal medicine, and ‘Jamu’. Phytopharmaca is clinically based herbal medicine and has complete clinical trial results for effectiveness and safety. Standardized herbal medicines (scientifically herbal medicine) have been tested for efficacy and toxicity but have not been clinical tested in patient. Jamu, on the other hand, is empirically based herbal medicine. Jamu has not undergone due diligence, and the used relying on the experiences of generations and societal traditions [29]. This classification explains that *jamu* is not just a drink but jamu is herbal medicine based on experience. *Jamu* has been practiced for centuries in society. It's still viral for maintaining health and treating diseases, and it's believed to be safe from chemical drugs. Sometimes, the peddlers of this herbal medicine went around in a region carrying the herbs they were selling. Hence, they were often called *jamu gendong* [19].

*Jamu gendong* is sold in baskets and carried around. It’s the traditional way to sell *jamu* [52]. *Jamu gendong* can still survive due to various methods or strategies used by *jamu gendong’s* sellers. The persistence of *jamu gendong* is due to its low and affordable price. Sales are carried out regularly door to door (from house to house), and the ingredients are still natural to make buyers sure of the quality. Eight (8) herbal ingredients that must be sold by sellers of *jamu* or *jamu gendong*, among others: *beras kencur* (a mixture of rice and *Kaempferia galanga* L which functions to treat body aches and as a tonic), *kunyit asem*, betle leaves, *temulawak* (*Curcuma xanthorriza* L to increase appetite), *cabepuyang* (a mixture of boiled *Piper retrofacrum, Curcuma xanthorriza* L and *Curcuma aeruginosa*, as an anti-inflammatory), kudu-laos (decoction of *Morinda citrifolia* and *Alpinia galanga*, to lower blood pressure), sinom (almost the same as *kunir asem* but the consistency is thinner due to less turmeric) and paitan [53]. Breastfeeding mothers can choose and buy one of these herbs according to their needs or health complaints. *Kunyit asem* or *sinom* is bought when there is menstrual pain. *Beras kencur* is bought when the appetite decreases or relieves cough and aches in the joints. *Paitan* to overcome allergies and itching. Betel leaves are selected to treat fluor albus. *Kudu-laos* to relieve sore throat and *uyup-uyup* to increase breastmilk production [54].

The types of herbs used by the respondents are uyub-uyub and papaya. Herbal research on breastfeeding mothers in Pringsewu and Madura districts, Indonesia, stated that the most used herb is Katuk leaves. Pringsewu is a district in Lampung Province [42, 55]. Madura is one of the islands in the province of East Java. Klaten is located in Central Java Province. Uyub-uyub is an icon herbal concoction from Central Java indicated as a breast milk booster [8]. The result shows differences in the types of herbs for breastfeeding mothers in each province of Indonesia.

Research on the use of herbs in Tanzania by Millinga et al. [28] stated that the most used herb for breastfeeding mothers is black pepper. Herbal research on breastfeeding mothers in Sierra Leone, Iraq, stated that the most used herb is *Cassia siberiana* [30]. Other types of herbs used in Iraq and Tanzania are also different, namely pumpkin seeds, raw groundnuts, ginger, lemon, cinnamon, garlic, raw cassava roots, lemongrass, aloe vera, clove, neem tree leaves, rabena, moringa, and tea bush [28, 30]. These herbs are also in Klaten, but the culture of drinking *uyub-uyub* for nursing mothers is more rooted in the Javanese community, especially the people of Klaten. It further strengthens the theory that the use of herbs is not only based on the affordability and accessibility of ingredients but also the cultural significance of each region or country [30].

The data showed three (3) significant benefits of using herbs for breastfeeding mothers: improving breast milk quality, making the body fit and healthy, and increasing appetite. However, some respondents stated that breastfed babies became fatter and more nutritious. The results of this study follow the results of Millinga et al. [28]. Herbal medicine is used to smooth breast milk, reduce pain, prevent stretch marks, and prepare the nipples for breastfeeding. Findings of indications for the use of herbs in breastfeeding mothers may vary depending on the health problems suffered by breastfeeding mothers in a country [28].

The data showed that the baby also experienced weight gain and the baby was healthier when the respondent consumed herbal medicine. It could happen because mothers perceive that their bodies’ condition affects their babies’ condition. Kent et al. [56] stated that the baby’s health condition was affected by the mother’s belief in the adequacy of her body’s milk. Mothers who feel their child is unhealthy or not growing perfectly believe that the breast milk produced by their body is insufficient for their baby. Mothers also observe whether the baby feels satisfied or not when breastfed. Mothers who think their babies are unsatisfied after breastfeeding will feel that the babies are not full and the milk is insufficient. This perception of inadequate breastfeeding causes mothers to make various efforts to overcome it, including using drugs (including herbal medicines) and visiting breastfeeding consultants [56].

Mattew et al. [57] showed that breast milk which is smooth due to consumption of herbs can cause weight gain for breastfed babies. Signs of adequacy of breast milk can also be measured from the parameters of the baby’s growth, the frequency of the baby’s urination, and the duration of the baby’s breastfeeding. The parameters of infant growth that are measured as an indicator of the adequacy of breast milk are the baby’s weight, length, and head circumference. Mehta [58] in post marketing surveillance stated that 46% of mothers who gave birth by Caesarean section experienced a very good increase in lactation after 3 up to 5 days of treatment with galactogogue. In the LBW infants, the mean weight is increased from 2.29 to 3.04 kg, while in the VLBW, the mean weight increased from 1.41 to 2.3 kg. The conclusion is that galagtogogue can increase lactation which results in baby’s weight gain.

The frequency of breastfeeding mothers consuming herbal medicine in this study was every day, every week, and every month. The majority use it every day. Respondents believed and expected that herbal medicine can provide optimal daily effects. The results of this study were also supported by Millinga et al. [28], which showed that 89.5% of respondents consumed herbal medicine daily. The use of herbal medicine every day indicated the respondent's belief in the efficacy and safety of herbal medicine. This belief is influenced by social culture. Trust in herbal medicines can grow because of social cultural influences from people who have the same interests or goals to obtain cheaper and more efficient treatment by using herbal medicines. Most people use herbal medicine from generation to generation because their parents passed it down. The stronger the socio-cultural support in society regarding the selection of herbal medicines, the more likely it is to choose traditional medicines, because the habits in the family are accepted by the community very easily [59].

Three respondents answered that they had experienced side effects from using herbal medicines. They believed herbs are safer than chemical drugs. Nausea and dizziness occurred in respondents who used *uyub-uyub*, jamu, and turmeric. Nausea and dizziness due to the use of sour turmeric may be due to the gastrointestinal effects of tumeric and tamarind and the antihypertensive effect of tamarind [40, 44]. Prastiwi reported dizziness in nursing mothers due to increased blood pressure from *uyup-uyup* [21]. If compared to the side effects of herbal medicine from the literature, the finding of side effects in this study are very mild. Some severe side effects, such as the side effects of paitan and betel leaves, were not experienced by the respondents. However, using these two herbs should be supervised by health professionals. The findings of the side effects of herbal medicines are conducted by Millinga et al. [28] which stated that the side effects felt by breastfeeding mothers who used herbs were usually nausea, abdominal pain, and diarrhea. Other studies also mention that the side effects of using herbs are significant because of the potential for toxicity in the presence of heavy metal contamination and the lack of scientific evidence supporting the safety of galactagogue herbs [60, 61].

Even though the incidence of side effects from using herbal medicines in breastfeeding mothers is low, the assistance of health workers in using herbal drugs is essential. Service can be provided by doctors, midwives, pharmacists, or other health workers who care for breastfeeding mothers. The assistance materials needed include the accuracy of how to obtain, how to use, how to store, and how to dispose of herbal medicines, how to be aware of side effects, and how to handle side effects.

The limitation of this study is not examined the factors that influence the choice of herbal medicines in breastfeeding mothers. The results showed that most of the benefits of herbs were facilitating breast milk so that the possibility of selecting herbal medicines was influenced by the smoothness of breast milk. However, it still requires further research. Research on the factors that influence behavior and the selection of herbal medicines in nursing mother needs to be recommended. The small sample size is also a weakness of this study because it was carried out only at three integrated service posts for infant and toddler health, so the results of this study relatively non-generalizable to the wider population. The future study is expected to use a larger sample and broadly in order to obtain better research results, more able generalized, can provide a more real picture of the use of herbal medicines in Klaten or Indonesia.

The explanation above stated that culture is a significant factor in how herbal medicine is used. Ignorance can harm herbs, and accurate analysis of small adverse effects is crucial for efficient application. Most knowledge about herbal medicine is coming from parents. Parents and families are encouraged to uphold the custom and culture of using herbal medicine and advisable to assess their knowledge in light of research on the safe and effective use of herbal remedies [59]. It is necessary to raise parental awareness of the efficacy and safety of herbal medications. In order to improve implementation, construction, as well as monitoring of herbal medicine’s usage, the family’s participation can also be increased through training in the safe use of herbal medicines.

In addition, it is also necessary to implement a strategy of integrating the use of herbal medicine with modern medicine. The integration program of herbal medicine with modern medicine has been initiated by the Ministry of Health of the Republic of Indonesia. Seven (7) steps to integrate traditional medicine into the health service system, namely the formulation of a strategy for integration, Setting regulations for integration, setting standards of service and competence, training and education for conventional providers and practitioners of traditional medicine; integration of traditional/alternative medicine into the (formal) health system; build partnerships and networks with other countries to exchange information and experience; and Conducting research and development for scientific proof [62].

A series of regulations, ranging from the legislative level to the decision of the Minister of Health, have been made in an effort to include traditional medicine in the national health system. The government has the authority to supervise traditional medicine, regulate traditional medicine practitioners, organize alternative practices, and develop scientific-based herbs. The herbal medicine program is also designed to encourage the use of herbal medicine by formal health professionals. This program aims to provide a scientific basis for using herbs in health services, build a network where doctors can function as providers and researchers (two systems), and encourage the provision of safe, effective, and high-quality herbs for use in health services [62]. The herb scientization program also involves clinicians, pharmacists and other health professionals. Herbal scientization training was also conducted for the health workers who introduced the herbal scientization program, effectiveness and safety of herbal, herbal quality, how to mix herbs and their correct use as well as research on herbs. The training was carried out by the herb scientization program team centered at Balai Besar Penelitian Dan Pengembangan Tanaman Obat dan Obat Tradisional, Tawangmangu (the Center for Research and Development of Medicinal Plants and Traditional Medicine, Tawangmangu) [63].

In fact, the use of herbal medicine is not supported by health professionals. Information about herbal medicine is rarely given by health professionals such as the correct use of herbal medicine, its effectiveness and safety, the correct rules of use, awareness of side effects, and the proper way of handling side effects. Health profesionals should also be active in the integration of herbal medicine with modern medicine. The form of integration is health professionals should actively participate in educating nursing mothers about herbal remedies. Increasing the active participation of health professionals in the use of herbal medicine in nursing mothers can be done by providing training to health professionals. Herbal medicine scientific training is conducted on health professionals so that they become health workers who are certified herbal medicine scientific and competent in the field of traditional medicine [63]. Health professionals must assist breastfeeding moms in using herbal medications because herbal medicine is a component of implementing health initiatives. These health professionals’ engagement can also help to prove the efficacy and safety of herbal remedies, as well as to protect them so that they can be utilized scientifically and under more strict supervision [29].

To support the use of herbal medicines that are efficient and safe for nursing mothers, traditional health promotion programs need the involvement of health workers. The program is also one way of integrating herbal medicine with formal health services. Public health centers officially conduct this program under the district or city government. As of October 2016, the number of health centers that have provided traditional health care is 2,143 health centers or 21.97% of the target of 25% of the total number of 9,754 health centers in Indonesia. The distribution of the number of people trained in self-care ‘family medicine garden’ and acupressure is 919 people in 34 provinces. One thousand five hundred thirty-two (1,532) health centers, or 15.7% in 2015, have provided traditional health services. Puskesmas that organize traditional health care must meet three criteria, including health centers with health workers trained in traditional health services and health centers carrying out self-care of traditional health herbs and skills. This program did not involve practitioners of traditional herbal medicine. The executor is a modern health professional who has undergone traditional health service training [64].

However, in the future, traditional health practitioners with formal education must perform traditional health services. Some educational institutions have established traditional health education, such as the diploma degree program of Indonesian herbal medicine at the Health Polytechnic of the Ministry of Health Surakarta and the undergraduate program of Traditional Medicine at the Airlangga University, Surabaya. Since 2016, the number of traditional health workers in government-owned formal health services like community health centers and hospitals has increased. The next step involves establishing the standard of traditional health services in formal health facilities providing guidelines for traditional health workers to execute their duties. The Regulation of the Minister of Health of the Republic of Indonesia Number 37 of 2017 on Traditional Health Services Integration and the Regulation of the Minister of Health of the Republic of Indonesia Number 15 of 2018 on the Provision of Complementary Traditional Health Services will temporarily used as guidelines in the implementation of traditional health services in formal health services [65, 66].

As an illustration of the form of cooperation between traditional health practitioners and modern health practitioners is as follows: Integrated Traditional Health Services combines conventional and complementary health services, serving as complementary and substitute services in specific circumstances. Integrated Traditional Health Services are performed jointly by traditional health practitioners and other health workers for patient treatment. The health workers join to form an integrated traditional health team. Traditional health practitioners must sort and evaluate whether the Client’s condition can be overcome with Complementary Traditional Health Services or referred to modern health services. The health workers join together to form an integrated traditional health team. Traditional health practitioners must sort and evaluate whether the Client’s condition can be overcome with Complementary Traditional Health Services or referred to modern health services. Traditional health practitioners must use Traditional Medicines that have a distribution permit or made by themselves and not give or use drugs or medicinal chemicals, narcotics, psychotropics, and dangerous substances. Traditional health practitioners prohibit radiation actions, invasive actions, and actions that use medical tools. Patients who require radiation, invasive procedures, and procedures that require special health equipment must be referred to clinicians or modern medicine practitioners. Clinicians who receive referral patients from traditional health practitioners can communicate with the referring Traditional Health practitioner based on the patient's interests [65, 66]. This mini-survey need further study about factors related to the behavior of using herbal medicines in breastfeeding mother.

## Conclusion

The pattern of traditional medicine use in the Jogonalan, Klaten is dominated by the use of uyub-uyub as a breast milk booster with a frequency of daily use. Other herbal medicines used are papaya leaves, turmeric, and aromatic ginger. Another benefit was breastfeeding mothers’ health and fitness and breastfed babies’ health. Only three respondents (6.7%) experienced side effects, namely nausea and dizziness. Breastfeeding mothers can choose herbal medicines with an evidence-based benefit and safety profile by increasing knowledge and opening discussions with health professionals and using them under the supervision of health professionals. The role of parents and health professionals needs to be increased in preserving herbal medicine by increasing knowledge and monitoring the effective and safe use of herbal medicines.

## Data Availability

All relevant data and materal are available in the manuscript without limitation and supporting data.
